# Obstacles encountered during transradial angiography from after Radial Artery puncture to the aortic arch

**DOI:** 10.1186/2193-1801-2-365

**Published:** 2013-07-31

**Authors:** Satoru Iwasaki, Kazuhiro Yokoyama, Kinya Furuichi, Hiroshi Okada, Akira Ohkura, Koichi Ide, Katsutoshi Takayama, Toshiaki Taoka, Kimihiko Kichikawa

**Affiliations:** Department of Radiology, Higashiosaka City General Hospital, Nishiiwata 3-4-5, Higashiosaka, Osaka 578-8588 Japan; Department of Neurosurgery, Higashiosaka City General Hospital, Nishiiwata 3-4-5, Higashiosaka, Osaka 578-8588 Japan; Department of Radiology and Interventional Neuroradiology, Ishinkai Yao General Hospital, 1-41 Numa, Yao, Osaka 581-0036 Japan; Department of Radiology, Nara Medical University, 840 Shijo-cho, Kashihara, Nara 634-8522 Japan

**Keywords:** Transradial angiography, Radial artery, Superficial brachial artery, Aortic arch anomalies

## Abstract

**Objective:**

To elucidate the key points for safe performance of transradial angiography.

**Conclusions:**

Transradial angiography can be performed safely if attention is paid to the following points from after radial artery puncture to reaching the aortic arch: resistance during guide wire operation for sheath insertion after puncture; confirmation of the superficial brachial artery; guide wire resistance while guiding the catheter to the aortic arch; and aortic arch anomalies.

## Introduction

Transradial angiography (TRA) has a number of advantages compared with transfemoral angiography (TFA), including the fact that postoperative hemostasis can definitely be performed using hemostatic devices rather than manual compression, meaning that anticoagulant therapy need not be discontinued for angiography, and the patient is able to get up immediately after the procedure and is thus more comfortable (Al-Kutoubi et al. 1996; Cowling et al. 1997; Matsumoto et al. 2001; Iwasaki et al. 2002; Jo et al. 2010). It also has advantages compared with transbrachial angiography, including the fact that anticoagulant therapy need not be discontinued and that the puncture site in the TRA is distant from the median nerve, making it safer than puncturing the brachial artery, which is adjacent to the median nerve (Heenan et al. 1996). In coronary angiography, TRA is used not only for diagnosis but also for interventions (Otaki 1992; Kiemeneij & Laarman 1993), although few institutions use this approach for cerebral (Matsumoto et al. 2001; Iwasaki et al. 2002; Jo et al. 2010) or other angiographies (Al-Kutoubi et al. 1996; Cowling et al. 1997). Reasons for avoiding TRA include: the narrow diameter of the radial artery, which makes it difficult to puncture; unfamiliarity with the obstacles that can occur along the route to the aortic arch; and the fact that catheter operations in the aortic arch are different from those of TFA. Radiologists engaged in angiography should possess the knowledge required for the safe performance of TRA for angiographic procedures. Focusing on the second reason mentioned above, the objective of this paper is to describe cases selected from around 2700 TRAs for cerebral angiography we performed as illustrative examples, with the aim of contributing to the safe performance of TRA.

### 1. Sheath insertion

The right radial artery was preferred if the Allen test permitted use of both sides (Iwasaki et al. 2002). When the right forearm is set along the torso, the position for the angiographer is almost the same as in the case of a right transfemoral approach. If the right Allen test warned for disconnection between the radial and ulnal arteries, the examination was performed via the left radial artery. After local anesthesia (about 1 or 2 ml of lidocaine 1%), the puncture was performed using a 22G (0.9 mm) puncture needle at the area of 2–5 cm proximal to the radial styloid.

After successful puncture of the radial artery, resistance may be felt when advancing the guide wire (0.025 inch, 0.635 mm) in order to insert the sheath (17 cm length with side holes, Medikit, Japan; 4F or 6F, 6F for the intervention). In such a case, the cause, as described below, must be investigated by confirming the location of the wire tip under fluoroscopy and gently injecting contrast agent from the puncture needle.

#### Radial artery occlusion

**Figure 1:**
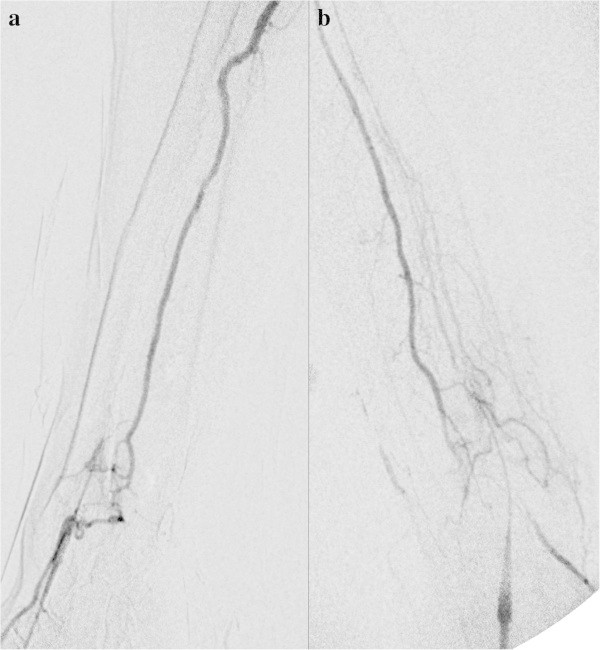
**Radial artery occlusion in a 74-year-old diabetic woman with occlusion of bilateral internal carotid arteries and the left vertebral artery. a**: DSA of the right forearm. **b**: DSA of the left forearm. Radial artery puncture was carried out successfully, but resistance was felt on inserting the guide wire near the puncture site in each side. Contrast was injected from the needles used to puncture each radial artery. The radial arteries on both sides are occluded, and the anterior interosseous artery, which had developed into a broad vessel via anastomoses, is contrasted. TRA was abandoned.

Even if the radial artery is occluded, it may be possible to feel a pulse and carry out puncture successfully. It is difficult, however, to continue the procedure (Figure 1).

#### Radial artery vasospasm

**Figure 2:**
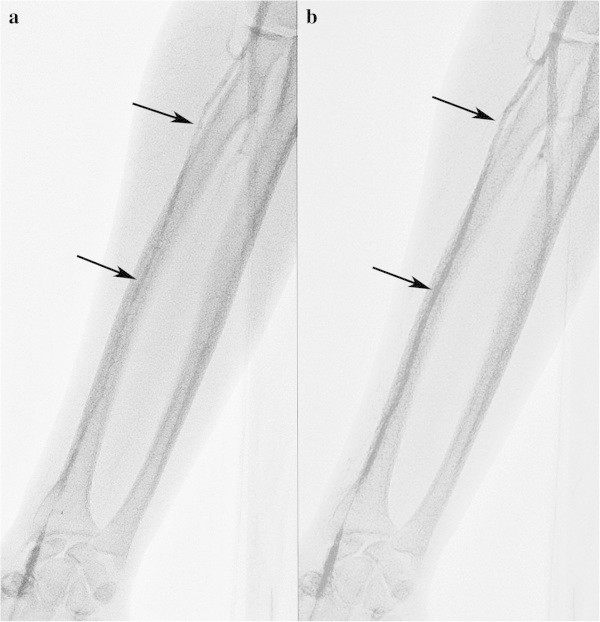
**Radial artery vasospasm in a 33-year-old woman with an arteriovenous malformation. a**: Resistance was felt when advancing the guide wire for sheath insertion. Following contrast agent injection via the puncture needle, radial artery vasospasm is observed. **b**: After injection of 0.25 mg of diluted nitroglycerin via the puncture needle, the radial artery can be seen to have dilated sufficiently to permit insertion of the sheath.

In young patients, simply puncturing the radial artery can cause vasospasm. A small amount of vasodilator (nitroglycerin 0.2–0.5 mg) should be injected via the puncture needle, and the sheath should be inserted after the artery has dilated (Figure 2).

#### Mistaken insertion of the guide wire into the recurrent artery

**Figure 3:**
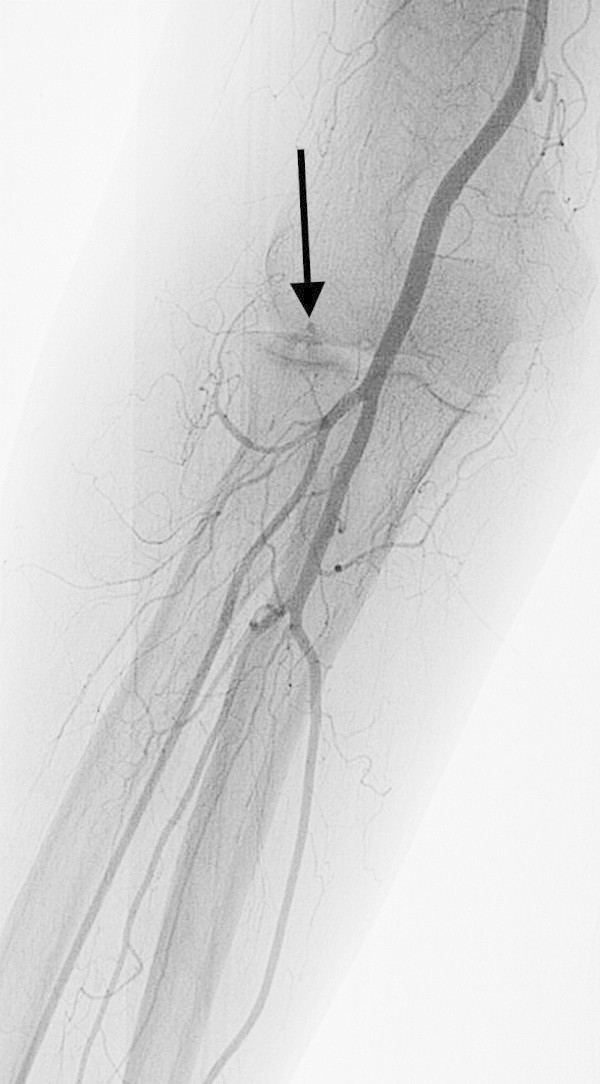
**Damage to the recurrent artery caused by the guide wire in a 72-year-old woman with a left vestibular Schwannoma.** Resistance was felt to wire operation for sheath insertion, and fluoroscopy showed that it was caught in the recurrent artery in the elbow region. After repeated reinsertions, the wire was successfully inserted into the brachial artery, and the sheath could be inserted. Subsequent contrast showed extravasation (arrow) and a subcutaneous hematoma, but manual compression prevented the hematoma from expanding. Ultrasound scanning at a later date confirmed that there was no formation of pseudoaneurysm or arteriovenous fistula. The damage probably occurred when resistance was felt during insertion of the wire. This type of situation can be expected if the wire is forcibly inserted into the recurrent artery, and treatment must be applied to ensure that the hematoma does not become too large.

If the tip of the wire is mistakenly inserted into a small artery such as the recurrent artery, ignoring the resistance to the wire and advancing it forcefully will damage the artery, producing a hematoma (Figure 3). If the wire consistently advances into the smaller artery, it is safer to advance it while referring to the map image produced by contrast agent injection.

#### Radial artery flexion

**Figure 4:**
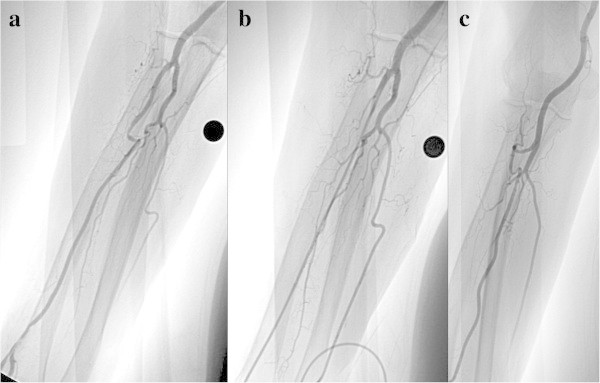
**Radial artery flexion. a** and **b**: A 72-year-old woman with an unruptured cerebral aneurysm. Resistance was felt when advancing the wire for sheath insertion, and contrast agent was therefore injected via the puncture needle, revealing sharp flexion of the radial artery **(a)**. When the wire (0.025 inch, diameter 0.635 mm) was advanced in line with the area of flexion, the flexure extended and it was possible to insert the sheath **(b)**. **c**: A 74-year-old woman with an unruptured cerebral aneurysm. Even when the guide wire for sheath insertion was passed through the area of flexion, the flexure did not extend, and sheath insertion was therefore discontinued. The SBA could not be identified, but the anastomotic branch between the SBA and the brachial artery was probably contrasted as the flexure of the radial artery.

With radial artery flexion due to arteriosclerosis, if the wire is passed through the site of flexion, the artery will extend along the course of the wire, permitting the sheath to be inserted without further action (Figure 4a, b). In the case of flexion at the anastomotic branch of the superficial brachial artery (SBA) described below, even if the wire does pass, the artery will not extend along the course of the wire, and sheath insertion must be abandoned (Figure 4c).

### 2. Passing the catheter through the brachial artery

**Figure 5:**
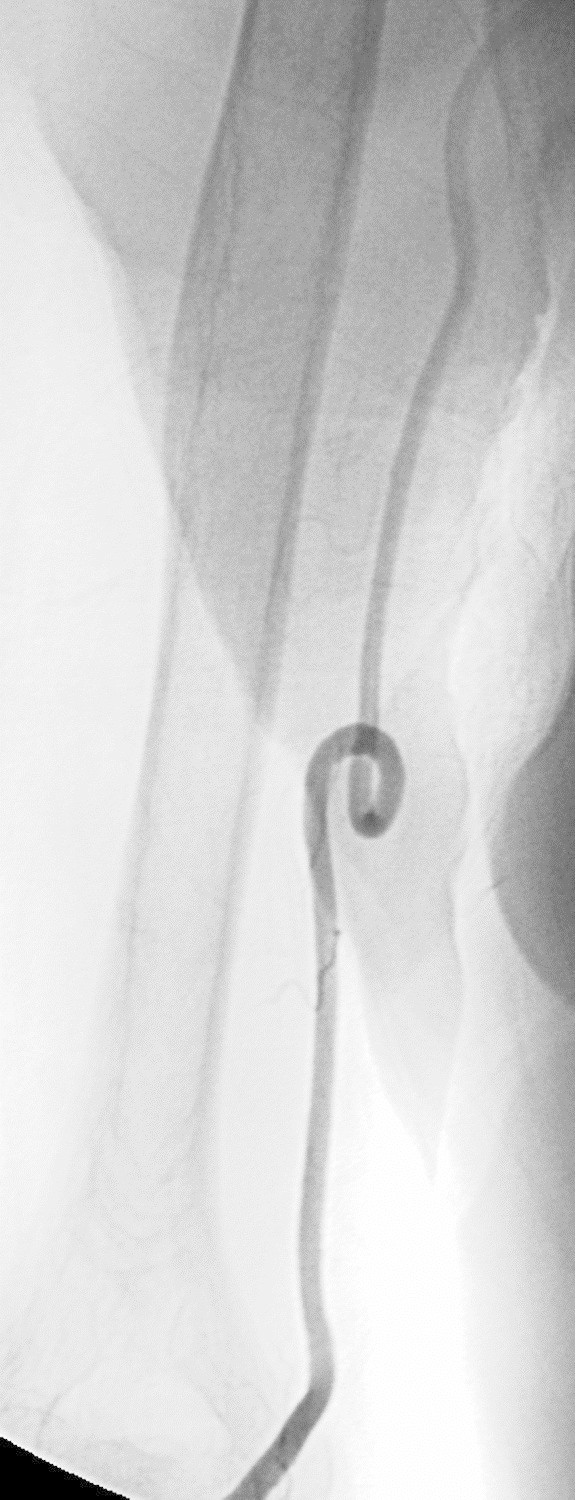
**Brachial artery flexion in a 68-year-old woman with a vestibular Schwannoma.** When the guide wire (0.035 inch, 0.889 mm) was passed through the flexure of the brachial artery, the flexure extended, enabling the catheter to be inserted and the examination to be performed.

After inserting the sheath, resistance to guide wire operation in the elbow or upper arm may be felt when advancing the guide wire (0.035 inch, 0.889 mm) to guide the catheter to the aortic arch. Possible reasons for this include: the wire having entered a small artery such as the recurrent artery or a muscular branch; sharp flexion of the brachial artery; occlusion between the brachial artery and the aortic arch; or encountering the SBA. Careful observation in the same way as during sheath insertion will enable the operator to realize if the wire has entered a branch of the brachial artery, and forceful operation must therefore be avoided. In most cases, flexion of the brachial artery is due to arteriosclerosis, and if a guide wire can pass through, it will extend along the course of the wire (Figure 5). The difference in right and left blood pressures is useful to predict occlusion along the route to the aortic arch.

### Superficial brachial artery (SBA)

**Figure 6:**
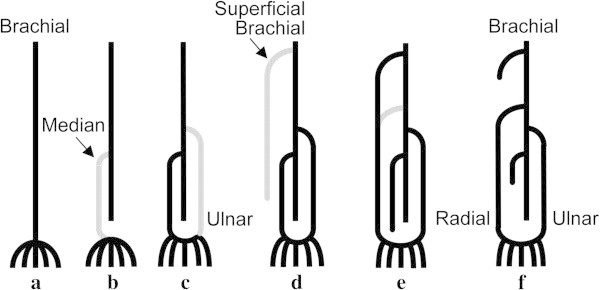
**Schematic diagram of the development of the brachial artery (modified from Lippert & Pabst (Lippert & Pabst**^1985^**)). a**: In embryos of body length 4–7 mm, the left and right 6^th^ (or 7^th^) cervical intersegmental artery develops as the axillary artery, becoming the brachial artery and then the interosseous artery, until it reaches the capillary plexus as finger arteries. **b**: The median artery arises from the interosseous artery and becomes the main supply for the finger arteries, while the periphery of the interosseous artery atrophies. **c**: When body length reaches 18 mm, the ulnar artery branches off the brachial artery and makes an anastomosis with the distal region of the median artery, forming the carpal arch. **d**: At body length 21 mm, the SBA is generated and reaches the wrist. **e**, **f**: When body length reaches 23 mm, the median artery degenerates, and the periphery of the SBA makes an anastomosis with the superficial volar arch. At the elbow, the anastomotic branch between the brachial artery and the SBA broadens, while the proximal side of the SBA atrophies, and the distal side becomes the radial artery.

This has been reported in anatomical studies with frequencies of 10% (Adachi 1928) and 13% (Lippert & Pabst 1985), but in the authors’ experience of angiography it has been 6.7%. If the SBA is extremely narrow, vasospasm occurs when the sheath is inserted, conceivably preventing its identification (Figure 4c). The arteries in the arm have many variants, and these are more easily understood in relation to their development, as shown in Figure 6. Because the SBA is not uncommon, it is safer to check for variants before catheter insertion. We perform digital subtraction angiography (DSA) of the elbow region before catheter insertion (3 mL of contrast agent from the sheath at 4 mL/s). The SBA branched from the axillary artery in 18.9% of our patients of SBA, the upper 1/3 of the brachial artery in 25.2%, the middle 1/3 in 37.8%, and the lower 1/3 in 12.6% (it could not be identified in 5.5%).

#### SBA without anastomosis (58.9% of SBAs investigated)

**Figure 7:**
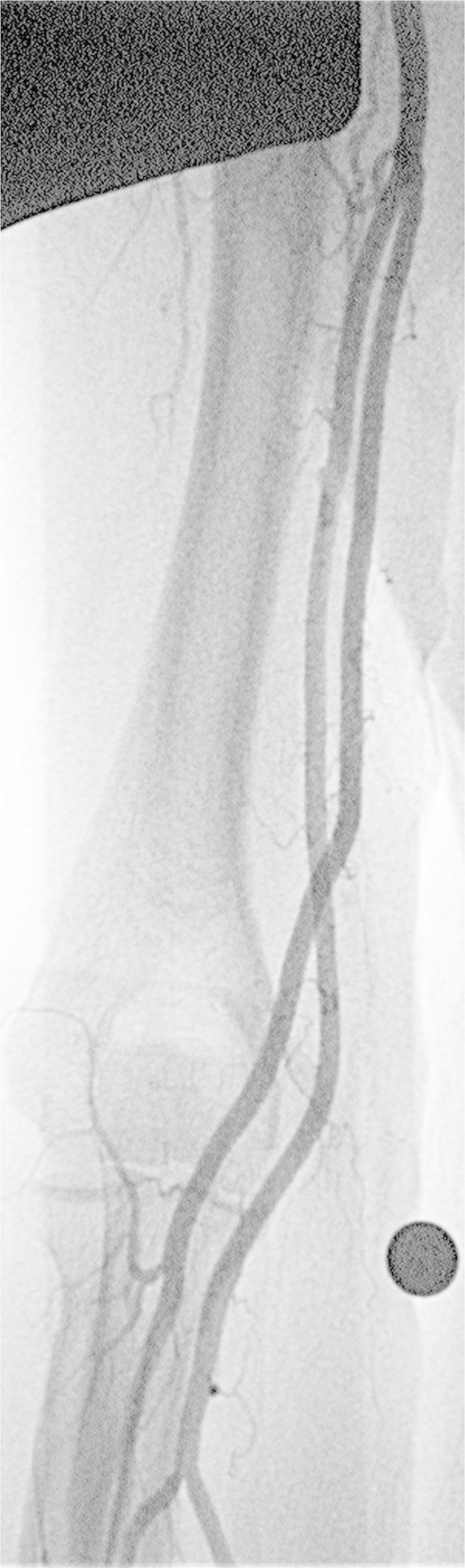
**SBA without anastomosis in a 51-year-old woman with a meningioma.** The radial artery branches off from a point in the central third of the brachial artery. The SBA persisted without formation of an anastomosis between the SBA and the brachial artery. If the SBA is as broad as it is in this case, the procedure can be continued without interruption, but if it is narrow, then it should be continued after interarterial infusion of vasodilator at this site.

The SBA is of almost the same diameter as the radial artery, so catheter insertion is possible (Figure 7). If the ulnar artery is predominant and the SBA is narrow, then catheter operation may cause vasospasm, so vasodilator (nitroglycerin 0.2–1.0 mg) should be injected before catheter insertion. In so doing, the vasodilator should be mixed with diluted contrast agent and injected while confirming the distribution of the agent.

#### SBA with an anastomosis, SBA is used (31.3%)

**Figure 8:**
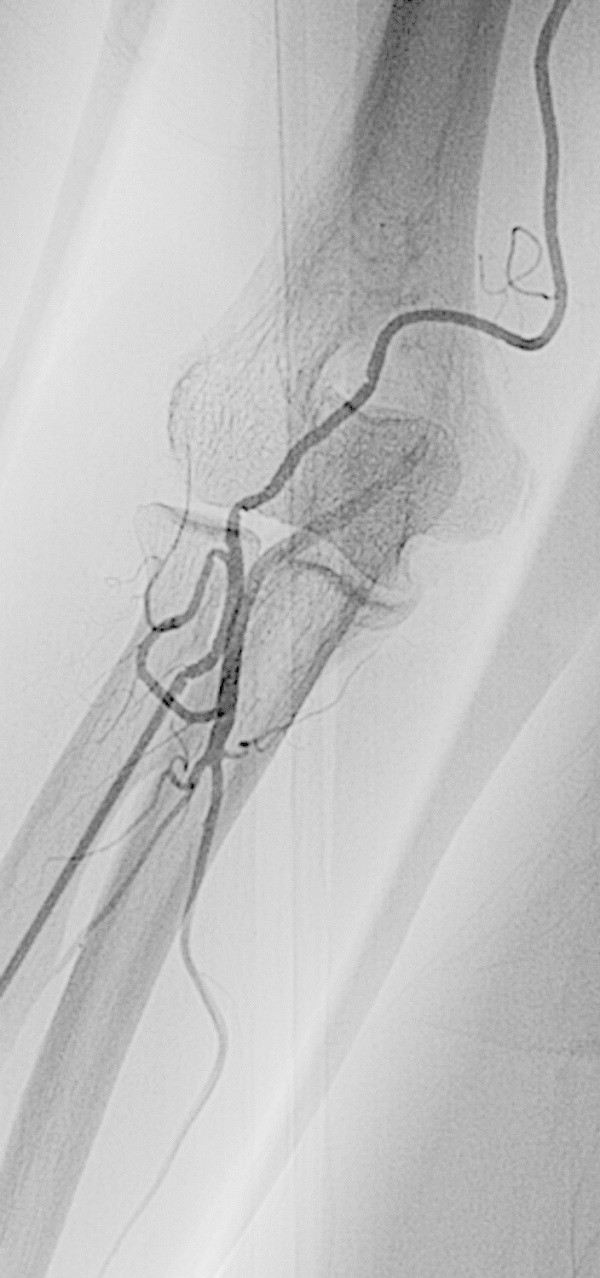
**SBA with anastomosis, SBA is used in a 69-year-old woman with unruptured cerebral aneurysm.** The SBA branches off a point in the central third of the brachial artery. The anastomosis forms a loop, meaning that the catheter could not be passed through it. The SBA is slightly narrower than the radial artery, so the examination was performed by inserting the catheter into the SBA after arterial infusion of vasodilator.

The wire will naturally enter the SBA, but since it is narrower than in the absence of the anastomosis, a vasodilator is often required (Figure 8).

#### SBA with an anastomosis, anastomosis is used (6.1%)

**Figure 9:**
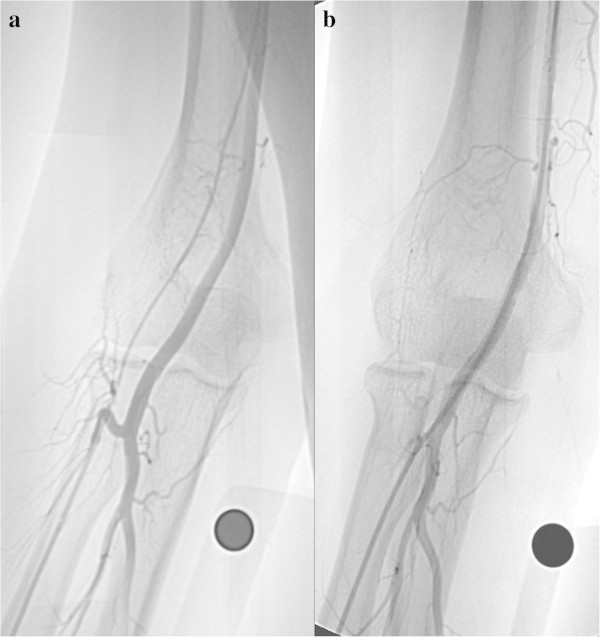
**SBA with anastomosis, anastomosis is used in a 48-year-old woman with a meningioma. a**: The anastomosis is broadly developed, whereas the SBA is narrow. **b**: The examination was performed by inserting the catheter into the brachial artery via the anastomosis.

If the anastomosis is broad and there is no sharp flexion, the catheter can be inserted into the brachial artery via this anastomosis (Figure 9).

#### SBA with an anastomosis, neither one can be used (3.7%)

**Figure 10:**
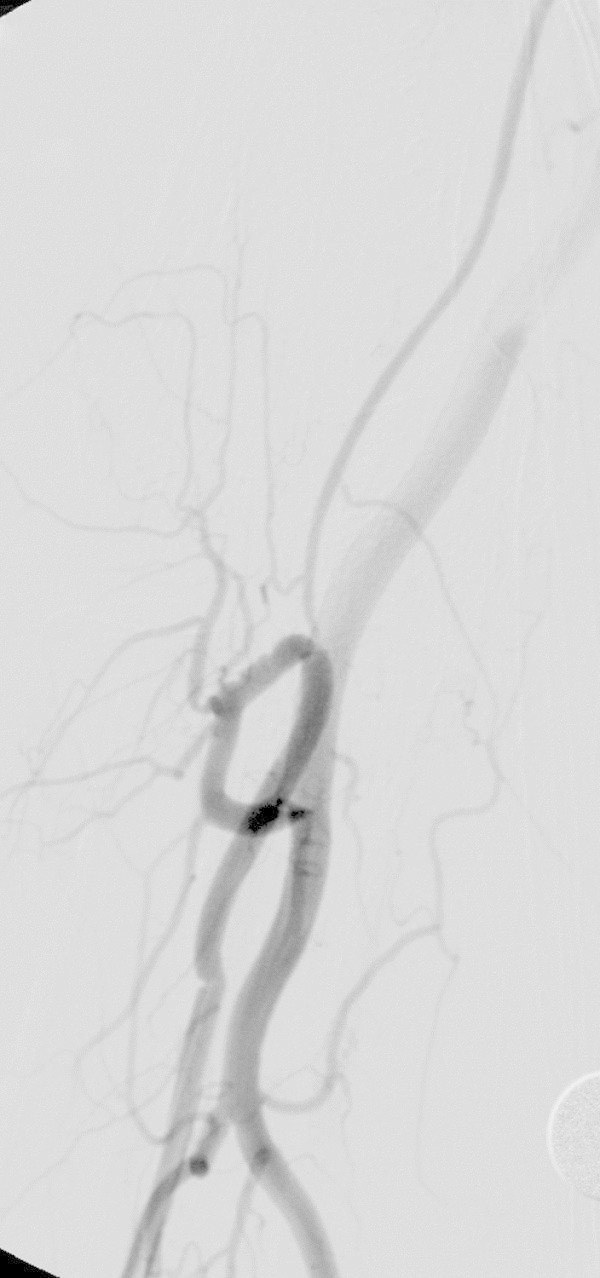
**SBA with anastomosis, neither SBA nor anastomosis can be used in a 61-year-old woman with an unruptured cerebral aneurysm.** Catheter insertion was not possible because the SBA was narrow. The anastomosis is broad but forms a loop, making it impossible to use for catheter insertion into the brachial artery. Angiography was switched to the left radial artery approach.

If the SBA is narrow and there is sharp flexion of the anastomosis, forming a loop, catheter insertion should be abandoned (Figure 10).

### 3. Aortic arch

The most frequently encountered anomalies of the aortic arch are aberrant right subclavian artery and right aortic arch.

#### Aberrant right subclavian artery

**Figure 11:**
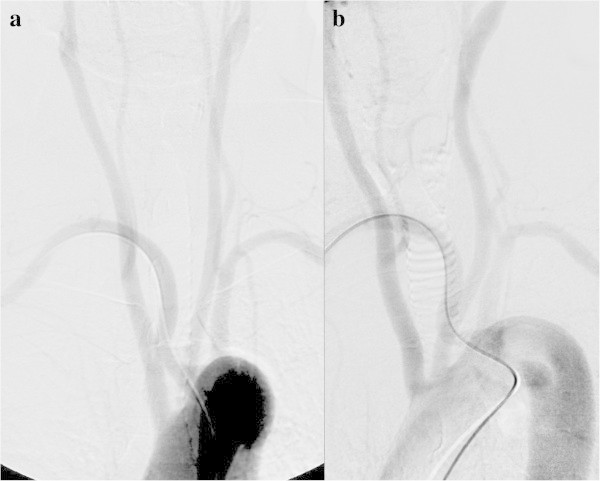
**Aberrant right subclavian artery in a 55-year-old woman with an unruptured cerebral aneurysm. a**: Arch aortography, frontal view. **b**: Left anterior oblique view. When the catheter was inserted into the aortic arch, it was found that the patient had an aberrant right subclavian artery with its origin on the left of the trachea. In most cases, the right vertebral artery branches off the right subclavian artery, but it may also branch off the right common carotid artery, as in this patient. Thus, when inserting the catheter into the right common carotid, it is necessary to check for the presence of the vertebral artery and avoid damaging it.

This has a reported frequency of 0.5% (Haughton & Rosenbaum 1974), the same rate seen in our patients. The right subclavian artery is known to branch off the aortic arch on the left side of the trachea. In TFA, the procedure may conclude without it having been noticed, but in TRA it must be thoroughly understood (Figure 11).

#### Right aortic arch

**Figure 12:**
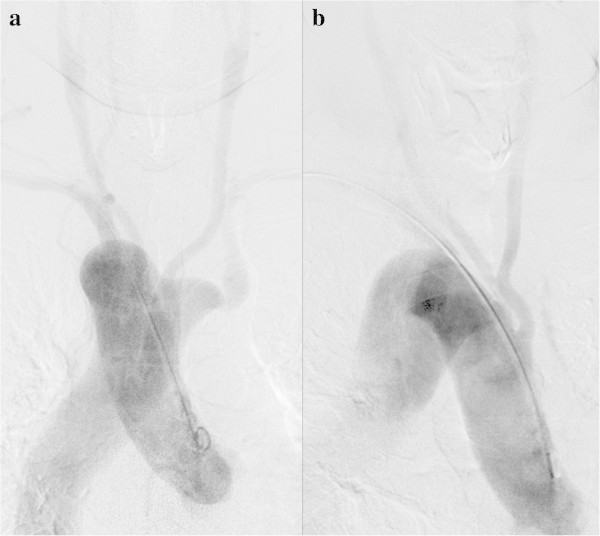
**Right aortic arch with aberrant left subclavian artery in a 61-year-old man with an unruptured cerebral aneurysm. a**: Arch aortography, frontal view. **b**: Right anterior oblique view. The left common carotid artery, right common carotid artery, and right subclavian artery branch off the aortic arch in that order, finally wrapping around the posterior side of the trachea and esophagus with a protruding Kommerell’s diverticulum, which is the vestige of the left aortic arch. Stenosis and post-stenotic dilatation of the aberrant left subclavian artery are visible beyond Kommerell’s diverticulum.

The rate was 0.02-0.06% in a report (Haughton & Rosenbaum 1974), and 0.11% in our patients. There are three variants: right aortic arch with aberrant left subclavian artery (Figure 12), right aortic arch with mirror-image branching of the arch vessels, and right aortic arch with isolation of the left subclavian artery (Stewart et al. 1966). The third one is extremely rare, and 98% of patients with mirror-image branching have cyanotic congenital heart disease (Stewart et al. 1966).

## Conclusion

We have presented illustrative examples of the obstacles that may be encountered along the route from after radial artery puncture until the catheter reaches the aortic arch during the performance of TRA. TRA can be performed safely if attention is paid to resistance during wire operation, confirmation of the SBA, and aortic arch anomalies.
